# Hospitalisation in an emergency department short-stay unit compared to an internal medicine department is associated with fewer complications in older patients – an observational study

**DOI:** 10.1186/s13049-017-0422-9

**Published:** 2017-08-15

**Authors:** Camilla Strøm, Talie Khadem Mollerup, Laurits Schou Kromberg, Lars Simon Rasmussen, Thomas Andersen Schmidt

**Affiliations:** 1 Department of Emergency Medicine, Holbaek Hospital, University of Copenhagen, Holbaek, Denmark; 2 Department of Anaesthesia, Centre of Head and Orthopaedics, Rigshospitalet, University of Copenhagen, Copenhagen, Denmark; 3 Institute of Clinical Medicine, Faculty of Health and Medical Sciences, University of Copenhagen, Copenhagen, Denmark; 4 Department of Anaesthesiology, Holbaek Hospital, University of Copenhagen, Holbaek, Denmark

**Keywords:** Emergency department short-stay units, Adverse events, Geriatric emergency medicine, Elderly patients, Accelerated care, Alternative hospitalisation strategies

## Abstract

**Background:**

Older patients are at particular risk of experiencing adverse events during hospitalisation.

**Objective:**

To compare the frequencies and types of adverse events during hospitalisation in older persons acutely admitted to either an Emergency Department Short-stay Unit (SSU) or an Internal Medicine Department (IMD).

**Methods:**

Observational study evaluating adverse events during hospitalisation in non-emergent, age-matched, internal medicine patients ≥75 years, acutely admitted to either the SSU or the IMD at Holbaek Hospital, Denmark, from January to August, 2014. Medical records were reviewed by independent assessors to detect adverse events according to predefined criteria. The primary outcome was the proportion of patients with an adverse event during and within 30 days after hospitalisation. Secondary outcomes included 90-day mortality, subtypes of adverse events, and timing of adverse events. Adjusted analyses were conducted to correct for potential confounders.

**Results:**

Four-hundred-fifty patients, 225 patients in each group, were included. Adverse events were found in 67 (30%) patients in the SSU-group and 90 (40%) patients in the IMD group (Odds Ratio (OR) 0.64 (95% Confidence Interval (95% CI) 0.43–0.94, *p* = 0.02). The result was unchanged in an analysis adjusted for age, Charlson Comorbidity score, and sex. We found no significant difference in 90-day mortality (OR 0.75, 95% CI 0.41–1.38, *p* = 0.36). The most common adverse events were transfer during hospitalisation, unplanned readmission, and nosocomial infection.

**Conclusions:**

Adverse events of hospitalisation were significantly less common in older patients acutely admitted to an Emergency Department Short-stay Unit as compared to admission to an Internal Medicine Department.

## Background

Globally, a recent development in acute care has been the widespread implementation of Emergency Department (ED) short-stay units (SSUs). SSUs are believed to increase the flexibility of the ED services by accommodating patients that need more time consuming investigations or observation [1–3]. Moreover, many SSUs provide brief hospitalisation for patients with minor medical ailments; thus, SSUs prevent short-term stay patients from being transferred to in-patient services. Often, a time limit of maximum stay in the SSU is set to 24, 48 or 72 h [4].

There is a lack of evidence regarding the safety of providing care for older patients in SSUs [1]. A hospital stay is associated with a risk of adverse events (AEs), such as nosocomial infections, medication errors, falls, or specific complications as a result of invasive procedures [5, 6]. An AE may not only delay recovery or prolong a hospital stay [7], it may result in death or persisting disability after discharge [8]. Older patients are at a high risk of acquiring AEs during hospitalisation [9], and the consequences are typically more serious compared to younger patients [5]. In light of the massively expanding number of older persons, expected to overwhelm the in-hospital services globally [10], it is relevant to address benefits and harms of alternative hospitalisation strategies such as hospitalisation in SSUs.

The aim of this study was to compare the proportion of older patients experiencing AEs during and within 30 days after hospitalisation in a SSU versus an internal medicine department (IMD). We hypothesised that the proportion of patients with an AE would be lower in the SSU-group. Secondly, we assessed 90-day all-cause mortality, subtypes of AEs, and timing of AEs.

## Methods

This observational study was based on data from electronic patient records. The Danish Data Protection Agency (REG-54-2015) and the Danish Health Authority (3–3013-1074/1) approved the study.

### Setting

We assessed medical records of patients acutely admitted to either the SSU or the IMD at Holbaek Hospital, Denmark, from January 1st 2014 to August 7th 2014. Holbaek Hospital is a secondary referral hospital with a catchment area of approximately 270,000 persons. In the study period, the ED evaluated approximately 160 patients per day, of which 3–5 patients were referred from a primary care physician directly to evaluation in the SSU. Usually, another 5–7 patients were relocated from the ED to the SSU per day. Patients were, with very few exceptions, admitted to the IMD after initial assessment in the ED.

### Description of the SSU

In 2012, the SSU was established as part of the ED to facilitate accelerated care or diagnostics for selected patients [11]. The SSU is under clinical governance by the ED staff; however, a few members of the staff are dedicated to the SSU and have the overall responsibility of that facility’s services. The SSU has 8 separate patient rooms, accommodating 2 patients per room and an additional room with 6 chairs for daytime patients. The SSU has access to the ED’s point-of-care investigations, satellite radiology room, and satellite laboratory, thus all investigations are executed on the same terms as in the ED. This also includes diagnostic tests in the Department of Radiology on a fast-track basis, such as Computed Axial Tomography scans. Patients are only admitted to the SSU if a hospital stay under 72 h seems realistic according to the physician evaluating the patient in the ED. The key philosophy of the SSU is that no patients should be held in the unit unless treatment is on-going and diagnostic tests should be applied on a fast-track basis. Patients are encouraged to be up and about without assistance during the stay, and the use of indwelling catheters is discouraged. Physical therapists and occupational therapists train and optimise the patients’ level of functioning upon request.

### Description of the IMD

The IMD consists of seven wards defined by sub-specialities: geriatrics, cardiology, endocrinology and nephrology, gastroenterology, and pulmonology. Each ward accommodates 20 patients, mainly with diseases belonging to the spectrum within the sub-speciality. The IMD has a large outpatient service and the majority of the IMD-physicians are internal medicine specialists, who work in both the inward and the outpatient services. The main difference between the services offered by the IMD and the SSU is that the IMD does not provide point-of-care laboratory or fast-track diagnostics, unless a patient is urgently deteriorating.

### Participants

To be included in the study, patients had to be 75 years or older, acutely admitted to hospital for an internal medicine disease, and triaged non-emergent at the time of admission. Patients were triaged ‘non-emergent’ if they presented to the ED with an acute illness, but displayed normal vital signs and no critical symptoms, according to the ED’s triage stratification definitions [12]. First, eligible SSU patients were identified. Second, IMD patients were identified by matching with SSU patients by year of birth and date of admission.

### Outcome hierarchy and variables

The primary outcome was the proportion of patients with an AE. Secondary outcomes were 90-day all-cause mortality, the proportion of patients acquiring the different subtypes of AEs, and we also assessed timing of the AEs. Length-of-stay in hospital (LOS) was reported, but not considered an outcome, because short LOS is believed to be a goal of SSU hospitalisation.

### Data sources

Patients were identified by the hospital’s electronic chart system (provided in the local ‘OPUS system’ and the nationwide ‘E-journal system’), which also were used for collection of data. Information on mortality was provided through the OPUS system from the Danish Civil Registration System (DCRS). All persons residing in Denmark have a unique personal identification number generated by the DCRS, the registry records vital status (alive/dead/emigrated) of all persons and is updated within a week of a person’s change of status.

### Measurements

For baseline characteristics, data included age, sex, arterial blood pressure, pulse rate, temperature, respiratory rate, arterial oxygen saturation, use of supplemental oxygen, information about smoking, and alcohol intake, Charlson comorbidity index (CCI), use of daily medications, date and time of admission, and admission diagnosis (according to WHO international classification of diseases version (ICD-10)). At discharge, we recorded the time and date of discharge, and the ICD-10 discharge diagnosis. We reported ‘diagnosis mismatch’ defined as cases where the admission diagnosis was different from the discharge diagnosis. Diagnosis mismatch was recorded as it was considered to be a sign of unclear symptoms or diagnoses at admission.

For outcomes, AEs were recorded by presence of event (yes, no, subtype), and time of the event (date, time). The AEs were defined based on Brennan et al.’s list of AEs in the Harvard Malpractice study [6]. We defined AEs as “Presence of one of 18 predefined unintended injuries or events that was caused by medical management rather than a disease process” (subtypes are listed in Table 1). AE’s were classified as in-hospital events and post-discharge events. The post-discharge events were inappropriate discharge at time of discharge and unplanned readmission within 30 days after discharge. To detect AEs, each patient record was screened by two physicians (CS, TM, or LSK). The physicians reviewed the records independently to detect an AE. After individual assessments, the results were compared. Disagreements were resolved by discussion and consensus, in case of discrepancies that were not easily resolved, a senior physician was consulted (TAS). We recorded vital status at 90 days from admission and time of death, if relevant.

**Table 1:** Basic characteristics

	Short-stay unit	Internal Medicine Department
	*n* = 225	*n* = 225
Age, median [IQR]	82 [78–86]	82 [78–86]
Age groups, n (%)		
75–79 years	98 (43)	97 (43)
80–84 years	67 (30)	68 (30)
85 years or older	60 (27)	59 (26)
Male sex, (n, %)	99 (44)	110 (49)
Mean arterial pressure in mmHg, mean (SD)	95 (14)	92 (16)
Pulse rate in beats per minute, mean (SD)	80 (14)	80 (15)
Temperature in degrees Celsius, mean (SD)	36.9 (0.6)	37.0 (0.8)
Respiratory rate in breaths per minute, mean (SD)	18 (3)	18 (4)
Pulse oximeter oxygen saturation in percent, mean (SD)	97 (2)	97 (2)
Supplemental oxygen on admission, n (%)	33 (15)	48 (21)
Smoking status never/previous/active (n, %)	141/57/27 (63/25/12)	130/76/19 (58/34/8)
Alcohol intake higher than recommend *, (n, %)	24 (11)	12 (5)
Number of medications used daily, median [IQR]	6 [4–9]	7 [4–10]
Charlson Comorbidity Index, median [IQR]	2 [1–3]	2 [1–3]
Charlson Comorbidity Index in groups, n (%)		
0	37 (16)	30 (13)
1	53 (24)	52 (23)
2	60 (27)	48 (21)
3	34 (15)	40 (18)
4	13 (6)	22 (10)
≥ 5	28 (12)	33 (15)
Reason for hospital admission, n (%)		
Alcohol withdrawal syndrome	1 (0.4)	0 (0)
Allergy/allergic reaction	2 (0.9)	0 (0)
Anaemia	39 (17.3)	5 (2.2)
Asthma	2 (0.9)	0 (0)
Back pain	6 (2.7)	0 (0)
Chronic Obstructive Pulmonary Disease	7 (3.1)	13 (5.8)
Phlebitis and thrombophlebitis of deep vessels of extremities	49 (21.8)	0 (0)
Dehydration/volume depletion	4 (1.8)	8 (3.6)
Delirium	1 (0.4)	1 (0.4)
Diabetes	3 (1.3)	2 (0.9)
Diarrhoea of none- infectious origin	2 (0.9)	9 (4.0)
Electrolyte imbalance	3 (1.3)	11 (4.9)
Erysipelas	10 (4.4)	7 (3.1)
Tendency to fall	21 (9.3)	7 (3.1)
Heart failure	0 (0)	12 (5.3)
Infection of unknown origin or not classified	7 (3.1)	28 (12.4)
Atherosclerosis of extremities	0 (0)	2 (0.9)
Constipation	0 (0)	4 (1.8)
Pleural effusion	1 (0.4)	4 (1.8)
Pneumonia	21 (9.3)	45 (20.0)
Poisoning	0 (0)	3 (1.3)
Renal disease (acute and chronic, not urinary tract infection)	3 (1.3)	4 (1.8)
Suspicion of any malignancy	8 (3.6)	1 (0.4)
Syncope/collapse	2 (0.9)	14 (6.2)
Pulmonary embolism	2 (0.9)	2 (0.9)
Urinary tract infection	5 (2.2)	20 (8.9)
Vertigo/dizziness	5 (2.2)	8 (3.6)
Other	21 (9.3)	15 (6.7)

Basic characteristics at time of admission for acutely admitted older internal medicine patients (≥ 75 years) treated in a Short-stay unit or an Internal Medicine Department presented as frequencies with percentages for categorical data, median values with inter-quartile range [IQR], or mean with standard deviation (SD) for continuous data. *Alcohol consumption according to the Danish Health Authority recommendation i.e. maximum of 7 units per week if female and 14 units per week if male. Abbreviations: IQR = interquartile range; SD = Standard Deviation

### Study size

The most common AE in older hospitalised patients has been identified to be medication error, affecting 30–37% [7, 9, 13]. We assumed that 37% of the IMD-population would have acquired an AE and assumed a 33% relative difference between the two groups to be a relevant difference to asses. Thus, based on a type-1 error of 5% and a statistical power of 80%, a total of 450 patients should be included.

### Statistics

Data were analysed using SPSS (SPSS Statistics for Windows, Version 20.0. Armonk, NY: IBM Corp 2011). The association between treatment in a SSU and occurrence of an AE was examined by binary logistic regression; results were given as odds ratio (OR) with 95% confidence intervals (95% CI). Both crude and adjusted analyses were performed, possible predictor variables were defined as: age-group, CCI-group, and sex. Additionally, the types of AEs were divided into two groups: in-hospital AEs and post-discharge AEs, and the same crude and adjusted analysis as described above were performed. The OR estimates with 95% CIs were plotted for separate events in a forest plot. 90-day mortality was tested with binary logistics. The interobserver agreement was examined by kappa-statistics. Unpaired Student’s t-test was used for group comparison of continuous variables. *P*-values below 5% were considered statistically significant.

## Results

We screened a total of 833 patients’ hospital records for inclusion of 450 patients, 225 patients in each group. Baseline characteristics were similar with the exception of reason for admission; more patients in the IMD-group were admitted due to infections (Table 2).

**Table 2:** Outcomes

	Short-stay Unit	Internal Medicine Department		
			Odds ratio (95% CI)	*p*-value
	(*n* = 225)	(*n* = 225)		
Patients with one or more adverse events, n (%)	67 (29.8)	90 (40.0)	0.64 (0.43–0.94)	0.02
Number of events per patient, n (%)				
0	158 (70.2)	135 (60.0)		
1	48 (21.3)	63 (28.0)		
2	13 (5.8)	16 (7.1)		
3	2 (0.9)	8 (3.6)		
4	3 (1.3)	3 (1.3)		
5	1 (0.4)	0 (0.0)		
Type of adverse event, n (%)				
Medication error	2 (0.9)	2 (0.9)	1.00 (0.14–7.16)	1.00
Drug side effect	1 (0.4)	3 (1.3)	0.33 (0.03–3.20)	0.34
Transfer to intensive care unit	4 (1.8)	3 (1.3)	1.34 (0.30–6.05)	0.70
Transfer to other unit	39 (17.3)	41 (18.2)	0.94 (0.58–1.53)	0.81
Unplanned surgery	2 (0.9)	6 (2.7)	0.33 (0.07–1.64)	0.17
Injury due to invasive procedure	0 (0)	0 (0)	-	1.00
Gastrointestinal bleeding	2 (0.9)	0 (0)	-	0.50
Neurologic deficit	0 (0)	2 (0.9)	-	0.50
Unexpected death	6 (2.7)	6 (2.7)	1.00 (0.32–3.15)	1.00
Cardiac arrest	0 (0)	1 (0.4)	-	1.00
In-hospital fall	1 (0.4)	7 (3.1)	0.14 (0.02–1.14)	0.07
Nosocomial infection	4 (1.8)	11 (4.9)	0.35 (0.11–1.12)	0.08
Decubitus acquired in hospital	0 (0)	1 (0.4)	-	1.00
Thromboembolic event	2 (0.9)	0 (0)	-	0.50
Disturbances of fluid balance	1 (0.4)	8 (3.6)	0.12 (0.02–0.98)	0.05
Other adverse event	3 (1.3)	4 (1.8)	0.74 (0.17–3.38)	0.70
Inappropriate discharge^*^	3 (1.3)	1 (0.4)	2.96 (0.30–28.66)	0.62
Unplanned readmission^*^	27 (12.0)	35 (15.6)	0.74 (0.43–1.27)	0.28
90-day mortality, n (%)	21 (9.7)	27 (12.6)	0.75 (0.41–1.38)	0.36
Length of stay in hospital, median in hours [IQR]	25 [9–71]	93 [43–190]	MDIF (95% CI) 103 (69–138)	<0.001

Adverse events, mortality, and length of hospital stay for acutely admitted older internal medicine patients (≥ 75 years) in a Short-stay unit versus an Internal Medicine Department. Categorical outcomes are presented as frequencies with percentages and group comparisons with Odds Ratio (OR) and 95% Confidence Intervals (95% CIs). Length-of-stay is presented with median and inter-quartile range [IQR], and group comparison is presented by the mean difference (MDIF) with 95% CI. *P*-values are given for all comparisons. ^*^Thirteen patients died in-hospital, thus these were not at risk for this event; furthermore 8 patients died within 30 days after discharge without adverse event. The definitions of adverse events are described in Text Box 1

Sixty-seven patients (30%) in the SSU-group vs. 90 patients (40%) in IMD-group had AEs, 97 events in SSU group vs. 131 events in the IMD-group, Table 3.

**Table 3:** Timing of in-hospital adverse events for older patients treated in Short-stay Unit/Internal Medicine Department

*Total number of patients at risk*	*450*	*238*	*92*	*38*	*18*
Adverse events Time in days	Day 0–1	Day 2–5	Day 6–10	Day 11–20	Day >20
Medication error, *n* = 4	1/0	1/2	0/0	0/0	0/0
Drug side effect, *n* = 4	0/0	1/1	0/2	0/0	0/0
Transfer to intensive care unit, *n* = 7	0/0	1/2	2/0	1/1	0/0
Transfer to other unit, *n* = 80	25/19	9/13	5/5	0/4	0/0
Unplanned surgery, *n* = 8	0/3	0/1	1/0	1/0	0/2
Injury due to invasive procedure, *n* = 0	0/0	0/0	0/0	0/0	0/0
Gastrointestinal bleeding, *n* = 2	0/0	0/1	0/1	0/0	0/0
Neurologic deficit, *n* = 2	0/0	0/0	0/2	0/0	0/0
Unexpected death, *n* = 12	0/0	0/2	1/2	2/2	3/0
Cardiac arrest, *n* = 1	0/0	0/0	0/1	0/0	0/0
In-hospital falls, *n* = 8	0/0	0/2	1/2	0/0	0/3
Nosocomial infection, *n* = 15	2/2	1/2	0/2	0/3	1/2
Decubitus acquired in-hospital, *n* = 1	0/0	0/0	0/0	0/1	0/0
Thromboembolic event, *n* = 2	1/0	1/0	0/0	0/0	0/0
Disturbance of the fluid balance, *n* = 9	0/3	1/3	0/1	0/1	0/0
Other event, *n* = 7	1/2	1/1	0/0	1/0	0/1

Cross table of the timing and type of in-hospital adverse events for acutely admitted older internal medicine patients (≥ 75 years) treated in a Short-stay Unit or an Internal Medicine Department

Adverse events are listed in the left column followed by the total number of the events. The ‘Time in days’ represents the time from admission to the time of the adverse event, as detected in the patient’s hospital chart. The ‘Total number of patients at risk’ identifies the total number of patients alive and still admitted in the given time interval. Each adverse event is represented by the number of patients in the Short-stay Unit and the Internal Medicine Department, who experienced the adverse event per time interval (number in Short-stay Unit/number in Internal Medicine Department)

**Fig. 1:**
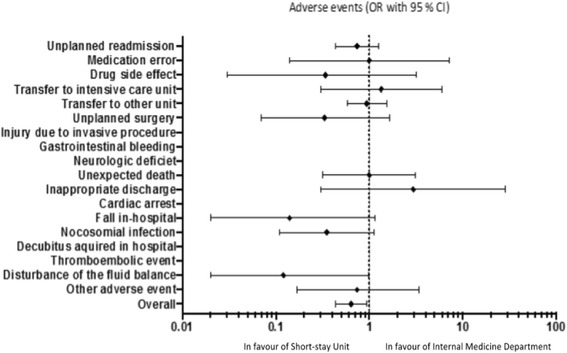
Forest plot of subtypes of adverse events occurring in acutely admitted older internal medicine patients (≥ 75 years) treated in a Short-stay unit versus an Internal Medicine Department. Differences between groups are expressed in Odds ratio (OR) with 95% confidence intervals (95% CI)

The OR for the primary outcome was 0.64 (95% CI 0.43–0.94, *p* = 0.02) in favour of the SSU. We found no significant difference in 90-day mortality SSU-group compared to the IMD-group, 21 (9%) vs. 27 (12%) died, (OR 0.75, 95% CI 0.41–1.38, *p* = 0.36). The most common AEs were transfer during hospitalisation, unplanned readmission, and nosocomial infection, Fig. 1. We found modest to good agreement between the observers (kappa = 0.71). Seventeen patients (8%) in the SSU-group vs. 17 patients in the IMD-group had diagnosis mismatch, (OR 0.34 (0.19–0.62), *p* < 0.001).

When dividing the AEs into events occurring in-hospital or post-discharge, the OR for in-hospital events was 0.58 (95% CI 0.37–0.90, *p* = 0.02) and OR for post-discharge events was 0.75 (95% CI 0.44–1.28, *p* = 0.33), in favour of the SSU.

When adjusting for potential explanatory factors (age, CCI, and sex), the OR was unchanged for the primary outcome, the in-hospital and the post-discharge events (adjusted OR for primary outcome: 0.64 (95% CI 0.42–0.94, *p* = 0.02); adjusted OR in-hospital events: 0.57 (95% CI 0.36–0.90, *p* = 0.02), and adjusted OR post-discharge events: 0.77 (95% CI 0.45–1.30, *p* = 0.33).

When adjusting for LOS and diagnosis mismatch, there was no longer a significant difference in the proportion with an AE; i.e. adjusted for LOS OR 0.81 (95% CI 0.46–1.43, *p* = 0.47); adjusted for diagnosis mismatch OR 0.71 (95% CI 0.68–1.06, *p* = 0.10).

For the timing of AEs, please see Table 4.

**Table 4:** Criteria for adverse events of hospitalisation

In-hospital events * Events that occur at any time during index hospital stay, regardless of whether the patient is transferred to another treatment unit later in the observation period. If clear documentation of suspicion of the disease or deficits existed in the admission note, it was not deemed an adverse event.* - Medication error - Drug side effect i.e. any adverse drug side effect or reaction - Transfer to intensive care unit - Unplanned transfer to another ward, department or hospital - Unplanned surgery i.e. any unplanned surgery - Injury due to invasive procedure i.e. any unplanned removal, injury, or repair of organ or structure during surgery, invasive procedure - Gastrointestinal bleeding - Neurologic deficit i.e. development of neurological deficit not present on admission - Unexpected death i.e. not an expected outcome of the disease during hospital stay - Cardiac or respiratory arrest - In-hospital fall - Nosocomial infection i.e. any hospital acquired infection or sepsis during index hospital stay - Decubitus acquired in hospital - Thromboembolic event i.e. any thromboembolic event including myocardial infarction, deep vein thrombosis, cerebrovascular accident, pulmonary embolism - Disturbances of fluid balance i.e. any episode of dehydration, overhydration, and electrolyte imbalances that was not present at time of admission - Any other undesirable outcomes (not covered by any of the other criteria) Post-discharge events - Inappropriate discharge defined as discharge in spite of presence of serious conditions that were not addressed during hospitalisation or time of discharge - Unplanned readmission defined as a return to hospital that led to unplanned admission within 30 days after discharge from index admission	

The majority of in-hospital events occurred within the 2 first days of admission (i.e. day 0 and day 1), in total 59 AEs. In-hospital falls were observed after 2 days, and delirium after 6 days. Most unplanned readmissions occurred 10 days after discharge: 2 within 48 h, 7 within 2–5 days, 12 within 6–10 days, 20 within 11–20 days, and 21 within 21–30 days.

## Discussion

We found that the proportion of patients acquiring an AE of hospitalisation was 10% lower in patients treated in a SSU compared to age- and triage matched patients treated in an IMD. The overall finding was mainly driven by in-hospital events.

The main strengths of our study are that we used a comparator, addressed potential confounding effects by matching and adjusting for possible predictor variables. AEs were assessed retrospectively but data were collected from electronic systems, in which patient data were entered prospectively. To minimise the risk of assessor bias, two independent physicians screened each patient record. We found modest to good agreement between assessors [14].

The main limitation of this study is the retrospective assessment of AEs. Despite that retrospective and prospective methods may identify similar rates of AEs [15], it is inevitable that our results could be influenced by unknown confounders, selection bias, or reporting bias. The CCI scores were similar in the groups indicating comparable morbidity, but we were not able to assess complex geriatric domains such as frailty, disability, or functional status, which in some previous studies have been found to be predictors of AEs [7, 16–20]. Nor were we able to incorporate frailty measures into a matching model, e.g. propensity score matching. This study was conducted in a single hospital, which may impair the external validity of findings. Lastly, the subtypes of AEs differed substantially and were not equally important, combining the events into a single outcome can be questioned; however, we did find that all but 2 events were more common in the IMD-group.

Most of the events occurred within the first 48 h after admission; this may indicate that patients had problems that were not addressed or overt at time of admission. In-hospital falls were observed after 2 days, and delirium after 6 days and that may reflect an increased risk of functional decline during hospitalisation. In-hospital transfer was more common in the SSU, which could be explained by the time limit for stay in the unit.

We found an association between AEs and both diagnosis mismatch and LOS, but a cause-effect relationship cannot be determined in the current study due to the observational design. AEs can lead to longer hospital stays [7]; however, short and effective hospital stay was partly the goal of SSU-hospitalisation; potentially, this model may lead to better patient outcomes by reducing time of exposure to AEs and preventing functional decline, which is particularly important for older patients. In fact, implementation of fast-track principles has been extensively studied in elective surgical populations with improved patient outcomes [21–23]. Acutely admitted older patients often present with ill-defined symptoms and more than one problem [24]. Mismatch between admission and discharge diagnosis may represent unclear symptoms or diagnoses, on the other hand it may indicate that patients encounter additional sickness or disabilities during the hospital stay, which may even be caused by AEs.

We found a frequency of AEs similar to those reported in previous studies [5, 9]. Some of the difference between the two groups may be explained by differences in underlying illness rather than as a consequence of the health care process. The groups differed with regard to reason for admission; this reflects a difference in case-mix between the settings, and the comparison between the groups should be interpreted with caution. However, there may be beneficial effects of SSU hospitalisation as described earlier. Previous studies comparing various SSUs using condition-specific protocols with traditional hospitalisation for adult internal medicine patients have indicated that SSU improve systems effectiveness by reducing the LOS for patients, optimize utilisation of health care, and reduce expenses [3, 25–27]; however, the body of evidence regarding patient outcomes are at this point sparse and the quality of previous studies graded low [1]. Randomised trials evaluating the effectiveness and safety of SSUs are needed to clarify the potential benefit of SSU for acutely admitted older patients.

## Conclusion

Adverse events of hospitalisation were significantly less common in patients 75 years or older acutely admitted to a short stay unit as compared to admission to an Internal Medicine Department.

## Abbreviations

AE: Adverse Events
CCI: Charlson Comorbidity Index
CI: Confidence Interval
ED: Emergency Department
IMD: Internal Medicine Department
LOS: Length Of Stay
OR: Odds Ratio
SSU: Emergency Department Short-stay Unit

## References

[1] Galipeau J, Pussegoda K, Stevens A (2015). Effectiveness and safety of short-stay units in the emergency department: a systematic review. Acad Emerg Med.

[2] Daly S, Campbell DA, Cameron PA (2003). short-stay units and observation medicine: a systematic review. Med J Aust.

[3] Downing H, Scott C, Kelly C (2008). Evaluation of a dedicated short-stay unit for acute medical admissions. Clin Med.

[4] Damiani G, Pinnarelli L, Sommella L (2001). The short stay unit as a new option for hospitals: a review of the scientific literature. Med Sci Monit Int Med J Exp Clin Res.

[5] Thomas EJ, Brennan TA (2000). Incidence and types of preventable adverse events in elderly patients: population based review of medical records. BMJ.

[6] Brennan TA, Leape LL, Laird NM (2004). Incidence of adverse events and negligence in hospitalized patients: results of the Harvard medical practice study I. Qual Saf Heal Care.

[7] Baker GR, Norton PG, Flintoft V (2004). The Canadian adverse events study: the incidence of adverse events among hospital patients in Canada. CMAJ.

[8] de Vries EN, Ramrattan MA, Smorenburg SM (2008). The incidence and nature of in-hospital adverse events: a systematic review. Qual Saf Health Care.

[9] Long SJ, Brown KF, Ames D, Vincent C (2013). What is known about adverse events in older medical hospital inpatients? A systematic review of the literature. Int J Qual Heal Care.

[10] Cowling TE, Soljak MA, Bell D, Majeed A (2014). Emergency hospital admissions via accident and emergency Departments in England: time trend, conceptual framework and policy implications. J R Soc Med.

[11] Petersen DB, Schmidt TA (2013). Quick diagnostic unit integrated in an emergency department setting reduces medical admissions – an observational study. J Hosp Adm.

[12] Skriver C, Lauritzen MM, Forberg JL, Gaardboe-Poulsen OB, Mogensen CB, Hansen CL, Berlac PA. Triage quickens the treatment of the most sick patients [Article in Danish]. Ugeskr Laeger. 2011;173(40):2490-3. 21975184

[13] Wilson RM, Runciman WB, Gibberd RW (1995). The quality in Australian health care study. Med J Aust.

[14] Brennan P, Silman A (1992). Statistical methods for assessing observer variability in clinical measures. BMJ.

[15] Michel P, Quenon JL, de Sarasqueta AM, Scemama O (2004). Comparison of three methods for estimating rates of adverse events and rates of preventable adverse events in acute care hospitals. BMJ.

[16] Lefevre F, Feinglass J, Potts S (1992). Iatrogenic complications in high-risk. Elderly Patients Arch Intern Med.

[17] Steel K, Gertman PM, Crescenzi C, Anderson J (1981). Iatrogenic illness on a general medical Service at a University Hospital. N Engl J Med.

[18] Becker PM, McVey LJ, Saltz CC (1987). Hospital-acquired complications in a randomized controlled clinical trial of a geriatric consultation team. JAMA.

[19] Madeira S, Melo M, Porto J (2007). The diseases we cause: iatrogenic illness in a department of internal medicine. Eur J Intern Med.

[20] Thomas EJ, Studdert DM, Burstin HR (2000). Incidence and types of adverse events and negligent care in Utah and Colorado. Med Care.

[21] Bagnall NM, Malietzis G, Kennedy RH (2014). A systematic review of enhanced recovery care after colorectal surgery in elderly patients. Color Dis.

[22] Khan MA, Pandey S (2016). Clinical outcomes of the very elderly undergoing enhanced recovery programmes in elective colorectal surgery. Ann R Coll Surg Engl.

[23] Spanjersberg WR, Reurings J, Keus F, van Laarhoven CJ. Fast track surgery versus conventional recovery strategies for colorectal surgery. Cochrane Database Syst Rev. 2011:CD007635. doi:10.1002/14651858.CD007635.pub2. 10.1002/14651858.CD007635.pub2 PMC13061361 21328298

[24] Aminzadeh F, Dalziel WB (2002). Older adults in the emergency department: a systematic review of patterns of use, adverse outcomes, and effectiveness of interventions. Ann Emerg Med.

[25] Miller CD, Hwang W, Hoekstra JW (2010). Stress Cardiac Magnetic Resonance Imaging With Observation Unit Care Reduces Cost for Patients With Emergent Chest Pain: A Randomized Trial. Ann Emerg Med.

[26] Gaspoz JM, Lee TH, Cook EF (1991). Outcome of patients who were admitted to a new short-stay unit to rule-out myocardial infarction. Am J Cardiol.

[27] Jibrin I, Hamirani YS, Mitikiri N (2008). Maryland’s first inpatient chest pain short stay unit as an alternative to emergency room-based observation unit. Crit Pathw Cardiol.

